# The right ventricle: interaction with the pulmonary circulation

**DOI:** 10.1186/s13054-016-1440-0

**Published:** 2016-09-10

**Authors:** Michael R. Pinsky

**Affiliations:** 1 Department of Critical Care Medicine, University of Pittsburgh, Pittsburgh, PA USA; 2 Department of Anesthesiology, University of California, East Campus Office Building, MC 7651, 9444 Medical Center Drive, Room 3-048, La Jolla, San Diego, CA 92093 USA

## Abstract

The primary role of the right ventricle (RV) is to deliver all the blood it receives per beat into the pulmonary circulation without causing right atrial pressure to rise. To the extent that it also does not impede left ventricular (LV) filling, cardiac output responsiveness to increased metabolic demand is optimized. Since cardiac output is a function of metabolic demand of the body, during stress and exercise states the flow to the RV can vary widely. Also, instantaneous venous return varies widely for a constant cardiac output as ventilatory efforts alter the dynamic pressure gradient for venous return. Normally, blood flow varies with minimal changes in pulmonary arterial pressure. Similarly, RV filling normally occurs with minimal increases in right atrial pressure. When pulmonary vascular reserve is compromised RV ejection may also be compromised, increasing right atrial pressure and limiting maximal cardiac output. Acute increases in RV outflow resistance, as may occur with acute pulmonary embolism, will cause acute RV dilation and, by ventricular interdependence, markedly decreased LV diastolic compliance, rapidly spiraling to acute cardiogenic shock and death. Treatments include reversing the causes of pulmonary hypertension and sustaining mean arterial pressure higher than pulmonary artery pressure to maximal RV coronary blood flow. Chronic pulmonary hypertension induces progressive RV hypertrophy to match RV contractility to the increased pulmonary arterial elastance. Once fully developed, RV hypertrophy is associated with a sustained increase in right atrial pressure, impaired LV filling, and decreased exercise tolerance. Treatment focuses on pharmacologic therapies to selectively reduce pulmonary vasomotor tone and diuretics to minimize excessive RV dilation. Owning to the irreversible nature of most forms of pulmonary hypertension, when the pulmonary arterial elastance greatly exceeds the adaptive increase in RV systolic elastance, due to RV dilation, progressive pulmonary vascular obliteration, or both, end stage cor pulmonale ensues. If associated with cardiogenic shock, it can effectively be treated only by artificial ventricular support or lung transplantation. Knowing how the RV adapts to these stresses, its sign posts, and treatment options will greatly improve the bedside clinician’s ability to diagnose and treat RV dysfunction.

## Background

The traditional concept of right ventricular (RV) function is that it is of minimal consequence for overall cardiovascular homeostasis. Indeed, at rest and with normal lungs and pulmonary vasculature the right ventricle can be completely cauterized with no measurable change in cardiac output [1]. Furthermore, RV infarction often creates an akinetic right ventricle without also causing cardiogenic shock. If anything, the major impact of RV function is usually considered to be a decrease in left ventricular (LV) diastolic compliance if RV over-distention occurs, through the process of ventricular interdependence [2–4].

The primary role of the RV is to deliver all the blood it receives into the pulmonary circulation on a beat-to-beat basis without causing right atrial pressure to rise. The blood it receives is called venous return and in the steady state equals cardiac output. The rate of venous return is a function of the pressure gradient for flow back to the heart from the periphery and its resistance. The body alters this pressure gradient and resistance as needed to match venous return to metabolic demands. Since cardiac output is a function of metabolic demand of the body, during stress and exercise states the flow to the right ventricle can vary widely. Complicating this further, instantaneous venous return also varies widely for a constant cardiac output as ventilatory efforts alter intrathoracic pressure (ITP) and with it the dynamic pressure gradient for venous return [5]. Markedly increased RV end-diastolic volumes associated with transient increasing venous return during spontaneous inspiration will limit LV end-diastolic volume less for the same LV filling pressure owing to the associated ventricular interdependence [2, 3] and pericardial volume restraint [6]. Under normal conditions pulmonary vascular compliance is adequate and pulmonary vascular resistance reactive enough to accept markedly increasing amounts of pulmonary blood flow with minimal increases in pulmonary arterial pressure. Finally, most investigators studying RV function had presumed it was a weaker but similar pump to that of the left side of the heart and measures such as diastolic compliance and end-systolic elastance can be similarly defined [7, 8]. Superficially, the right ventricle appears to behave like the left ventricle in that increases in filling pressure increase RV stroke volume and “Starling” filling pressure to stroke volume curves can be created on a beat-to-beat basis over the ventilatory cycle [9]. But these studies never measured RV pressures and volumes simultaneously nor took into account pericardial pressure. When such dynamic pressure–volume analyses were finally performed, it became clear the right heart behaves very differently from the left. These differences and the RV response to increasing pulmonary outflow resistance for pulmonary vascular compliance form the basis of this review.

## RV filling

Tyberg et al. [10] measured pericardial pressure and right atrial pressure during acute fluid loading in patients prior to going on cardiopulmonary bypass. They showed that both right atrial pressure and pericardial pressure increased equally such that the transmural right atrial pressure (right atrial pressure minus pericardial pressure) remained unchanged. Pinsky et al. [11] subsequently duplicated these findings in post-operative cardiac surgery patients by examining the effect of changing ITP and end-expiratory lung volume by varying positive end-expiratory pressure (PEEP) on RV function. RV volumes were estimated by the ejection fraction technique of the thermodilution profile as the ratios of sequential reciprocals of the residual thermal signal following a right atrial cold saline bolus using a rapid response thermistor pulmonary artery catheter. They found that there was no relationship between transmural right atrial pressure and either RV end-diastolic volume or stroke volume. However, as the RV end-systolic volume increased the RV ejection fraction decreased, such that the relationship between RV end-diastolic volume and end-systolic volume was highly linear with a slope equal to the RV ejection fraction. These data support the hypothesis that the normal human right ventricle fills at or below its unstressed volume, such that RV end-diastolic volume changes occur without any change in RV diastolic wall stretch. Presumably, conformational changes in RV shape rather than stretch allow these volume changes to occur without measurable changes in transmural right atrial pressure (RV distending pressure). Since Starling’s law of the heart relates preload to end-diastolic wall stress, then RV preload, which is wall stretch, would remain constant as RV end-diastolic volume varied because preload is the pressure force, which arises with different compliances. In support of this hypothesis, several groups reported that there was a reverse linear relationship between RV end-diastolic volume and RV ejection fraction. If, as with the left ventricle, increased RV filling increased RV wall stretch, then one would expect RV ejection fraction to increase by the Frank–Starling mechanism [12–14]. The corollary to these findings is that if RV volume did alter RV preload, then the right ventricle was either hypertrophied with diastolic dysfunction or over distended as in acute cor pulmonale. Finally, since increasing RV end-diastolic pressures often reflect only pericardial distention and not change in RV distending pressure, the interpretation of increasing right atrial pressures as a measure of RV preload is problematic. Indeed, right atrial pressure can increase with decreasing RV dilation, as commonly occurs with increasing ITP associated with the application of PEEP.

Other implications of these data are that measures of right atrial pressure (or central venous pressure) could thus never be used to predict volume responsiveness [15] but an increase in right atrial pressure in response to fluid loading would be an excellent measure of impending RV failure once fluid resuscitation had exceeded the normal RV unstressed volume operating range. In support of this construct, Jardin et al. [16] measured RV volumes in patients with acute lung injury given fluid resuscitation. They found that if they restored RV volume to their baseline values with fluid resuscitation, cardiac output increased, whereas if further fluid resuscitation was done, cardiac output did not increase but right atrial pressure rose abruptly and a paradoxical diastolic leftward shift of the intraventricular septum developed. When simultaneous RV pressure and volume measures were made throughout the cardiac cycle, with RV pressures referenced to pericardial pressure, RV diastolic fill appeared to occur without any change in filling pressure and in some cases was associated with a fall in filling pressure [17, 18]. Thus, under normal conditions, absolute RV end-diastolic pressure is more a function of pericardial restraint, pericardial pressure, and ITP than of RV end-diastolic stretch.

## RV ejection

RV contraction is functionally different from LV contraction [19]. LV contraction causing decreases in LV volume primarily results from combined cross-sectional area reductions due to circumferential fiber shortening and twisting or “wringing” owing to oblique fiber shortening with longitudinal axis shortening. RV contraction on the other hand primarily occurs by longitudinal shortening and occurs in a peristaltic fashion starting with inflow track contraction and proceeding to RV mid-wall then RV outflow track (infundibulum) contraction with a timing difference of approximately 25–50 ms [20]. Thus, measures of RV peak systolic strain by echocardiography reflect a sensitive measure of RV systolic performance [21].

Contractile function can be assessed in a variety of ways. One can measure stroke volume, stroke work, ejection fraction, and velocity of circumferential fiber shortening. However, the most accurate measure of systolic function is time-varying elastance and end-systolic elastance, as a measure of the progressively increasing stiffness that the heart undergoes during contraction [22]. LV end-systolic elastance (Ees) is the slope of the end-systolic pressure–volume relationship and is approximated by the maximal LV systolic pressure to volume ratio at end-ejection. It is highly correlated with contractility and though the actual end-systolic pressure and volume will be a function of both LV preload and afterload, the slope of the line is independent of these variables. Although RV Ees can also be measured if one knows end-systolic RV pressure and volume, it does not describe RV contractility as much as systolic ventricular interdependence [23] because more than half of RV developed pressure comes from LV free wall contraction [24]. This explains why insertion of a LV assist device in a patient with combined acute LV failure and mild pulmonary hypertension often induces acute RV failure [25]. When pulmonary vascular reserve is compromised, as in pulmonary hypertension and LV failure, RV ejection is also compromised, initially causing right atrial pressure to rise in response to increased venous return and eventually to remain elevated even at rest. RV ejection is also compromised, initally causing right atrial pressure to rise in response to the decreased RV stroke volume and increased RV end-diastolic volume, causing venous return to decrease. If sustained, right atrial pressure will remain elevated at rest. This combined impaired RV ejection and increased right atrial pressure is also associated with a markedly decreased maximal cardaic output in response, limiting exercise tolerance and causing fluid retention.

Acute increases in RV outflow resistance, as may occur with acute pulmonary embolism and hyperinflation, will cause acute RV dilation and, by ventricular interdependence, markedly decreased LV diastolic compliance, decreasing LV stroke volume, cardiac output, and arterial blood pressure and rapidly spiraling to acute cardiogenic shock and death. Coupled with these findings is the reality that RV ejection is exquisitely dependent on RV ejection pressure [26]. Presumably, this is also the cause of backward LV failure causing RV failure. As LV systolic function deteriorates, stroke volume decreases owing to an increase in LV end-systolic volume. Clearly this must increase LV end-diastolic volume and filling pressures. If pulmonary vascular resistance is unchanged, the increase in left atrial pressure will be reflected back to pulmonary artery pressure, increasing RV afterload. Thus, the combined decreased LV contraction coupled with the increased pulmonary arterial pressure may lead to the commonly seen biventricular failure. Since this process usually happens gradually, fluid retention concomitantly occurs, producing peripheral edema as right atrial pressure rises. If LV failure occurs rapidly, as may occur with an acute coronary syndrome, then the pooling of blood in the lungs associated with acute cardiogenic pulmonary edema will also be associated with a relative hypovolemia. It is unclear if mean systemic filling pressure, the equilibrium stop flow pressure in the circulation, will also decrease in this scenario of acute LV failure despite the shift of blood from the peripheral to the central compartment. Concomitant with the induction of acute heart failure, profound increases in sympathetic tone also occur, increasing arterial resistance and decreasing venous capacitance. Thus, patients presenting with an acute coronary syndrome often display systemic hypertension, tachycardia, and pulmonary edema with elevated left- and right-sided filling pressures. The common clinical mistake is to interpret these findings as general volume overload and treat the pulmonary edema with a diuretic as opposed to an afterload-reducing agent. The diuretic will worsen the circulatory shock whereas the afterload-reducing agent will not. Examples of afterload reduction include using continuous positive airway pressure to abolish the negative swings in ITP, narcotics as sympathetolitics, and pharmacologic vasodilators (e.g., nitroglycerine).

Furthermore, most of the RV coronary blood flow occurs during systole, unlike LV coronary blood flow, which primarily occurs in diastole [27]. Thus, systemic hypotension or relative hypotension where pulmonary artery pressures equal or exceed aortic pressure must cause RV ischemia. Treatments here include not only reversing the causes of pulmonary hypertension but efforts to sustain mean arterial pressure higher than pulmonary artery pressure to maximal RV coronary blood flow. Clinically, this is usually done by the infusion of potent vasoconstrictor agents (e.g., norepinephrine).

Clinically, these findings carry a common end result. For cardiac output to increase, RV volumes must increase. If increasing RV volumes also results in increased filling pressures, then RV over-distention may occur, causing RV free wall ischemia. It is not clear at what pressure RV volumes become limited but this probably occurs at relatively low transmural pressures of ~10–12 mmHg. As mentioned above, however, if pericardial pressure is also increased, then right atrial pressure may be quite high without RV dilation. If relative systemic hypotension co-exists, then selective increases in arterial pressure will improve RV systolic function. Accordingly, fluid resuscitation, if associated with rapid increases in right atrial pressure, should be stopped until evidence of acute cor pulmonale is excluded [28]. Acute cor pulmonale is treated by improving LV systolic function, maintaining coronary perfusion pressure, or reducing pulmonary artery outflow impedance. Since more than half of RV systolic force is generated by LV contraction, through the free wall interconnection of fibers and not through stiffening or thickening of the intraventricular septum [24], efforts to increase LV contractility independent of maintaining coronary perfusion pressure are important. Since RV coronary perfusion primarily occurs during systole, maintaining coronary perfusion pressure greater than pulmonary artery pressure by the use of systemic vasopressor therapy is also indicted [27]. Finally, since increased RV afterload is a major limitation to RV ejection, efforts to minimize pulmonary vascular resistance and increase pulmonary vascular compliance are also beneficial.

## RV afterload

The right ventricle, as opposed to the left ventricle, ejects blood into a low-pressure, high-compliance pulmonary circulation. Although the absolute compliance of the pulmonary circulation is one-seventh that of the systemic circulation, it stores much less blood and has the ability to collapse pulmonary vessels as well as have them distend. Thus, the pulmonary circulation is capable of accommodating increased blood volumes without increasing pulmonary artery pressure as much as would occur on the systemic side if similar increases in flow were seen in the aorta. This greatly benefits RV systolic function during exercise. Despite being compliant, this circuit does pose resistance to the ejecting right ventricle as quantified by pulmonary arterial pressure. RV afterload is conceptually similar to LV afterload and is determined by the wall tension of the RV. Under normal conditions RV afterload is highly dependent on the distribution of blood flow in the lung, the degree of hyperinflation or increased alveolar pressure that may be present [29], and active increases in pulmonary vasomotor tone as may occur with inflammation and alveolar hypoxia.

Increases in lung volume independent of changes in pulmonary vasomotor tone can also alter RV function [30, 31]. With inspiration, the expanding lungs compress the heart in the cardiac fossa [32], increasing juxtacardiac ITP. Because the chest wall and diaphragm can move away from the expanding lungs, whereas the heart is trapped within this cardiac fossa, juxtacardiac ITP usually increases more than lateral chest wall ITP [33, 34]. This selective cardiac compressive effect is due to increasing lung volume. It is not affected by the means whereby lung volume is increased. Thus, both spontaneous [35] and positive pressure-induced hyperinflation [36–38] cause similar compressive effects on cardiac filling. If one measured only intraluminal LV pressure, then it would appear as if LV diastolic compliance was reduced because the associated increase in pericardial pressure and ITP would not be seen. When LV function is assessed as the relationship between end-diastolic volume and output, however, no evidence for impaired LV contractile function is seen [39] despite the continued application of PEEP [40]. These compressive can be considered as analogous to cardiac tamponade. This hyperinflation-induced impaired LV filling forms the basis for the recently completed clinical trial of pharmacologic lung reduction therapy using bronchodilator therapy [41]. By focusing on minimizing hyperinflation, the authors showed that exercise tolerance markedly improved and remained elevated over time.

In the aggregate, if RV failure occurs due to increased afterload or impaired contractility, then RV filling pressures rise and if mean systemic filling pressure does not rise proportionally, cardiac output falls because the pressure gradient for venous return decreases. Indirectly, this gives rise to the clinical observation that if fluid resuscitation causes right atrial pressure to rise, then the patient is probably not volume responsive because the normal response of a healthy individual to fluid resuscitation is to keep right atrial pressure constant as cardiac output increases owing to the increased pressure gradient for venous return to the heart.

## Ventriculo-arterial coupling

RV ejection of blood and input pulmonary arterial pressure and tone are tightly coupled. Clearly the cause of the pulmonary arterial pulse pressure is stroke volume. For the left side the mean arterial pressure and the magnitude of the pulse pressure rise for a given stroke volume are also a function of the input arterial resistance, compliance, and impedance [42]. As described above, RV Ees can be defined as the slope of the RV end-systolic pressure–volume relationship. RV end-systolic pressure is also a function of both RV stroke volume and a physical characteristic of the pulmonary arterial outflow tract. The greater the RV stroke volume for a given vascular tone and compliance, the greater the systolic pulmonary arterial pressure. However, systolic pulmonary arterial pressure is also a primary determinant of RV afterload. Thus, increases in systolic pulmonary arterial pressure for a constant preload and Ees will decrease RV stroke volume and increase RV end-systolic volume. The slope of the relationship between RV stroke volume and systolic pulmonary arterial pressure is called pulmonary arterial elastance (Ea). Thus, RV stroke volume not only is limited by but defines end-systolic pressure though arterio-ventricular coupling. Both Ees and Ea can be assessed at the bedside using combined RV pressure and estimates of RV stroke volume [43].

Prior studies have identified that maximal LV myocardial efficiency, defined as the amount of external work performed for myocardial oxygen consumed, occurs when systemic Ea is approximately one-half LV Ees [44] and shows a stronger dependence on systemic Ea than LV Ees [45]. Accordingly, LV Ea/Ees is a sensitive and independent estimate of the efficiency of the cardiovascular system [46]. A normally coupled cardiovascular system (Ea/Ees 0.5–1) is efficient and provides adequate stroke work. Uncoupling, defined as Ea/Es >1 or <0.5, can result from changes in LV Ees, Ea, or both [47]. Uncoupling reflects a reduction of the LV ejection efficiency that can promote LV energetic failure. Guarracino et al. [48] recently showed that septic shock patients display profound Ea/Ees uncoupling that may contribute to the observed LV dysfunction in sepsis. Presumably, this uncoupling occurs owing to a primary decrease in peripheral impedance and increase in peripheral arterial compliance [49]. Similar analysis can be done for the right ventricle. It is not clear if a similar optimal Ea/Ees ratio exists for the right ventricle. But many studies have shown that excess pulmonary Ea to RV Ees causes heart failure.

**Fig. 1:**
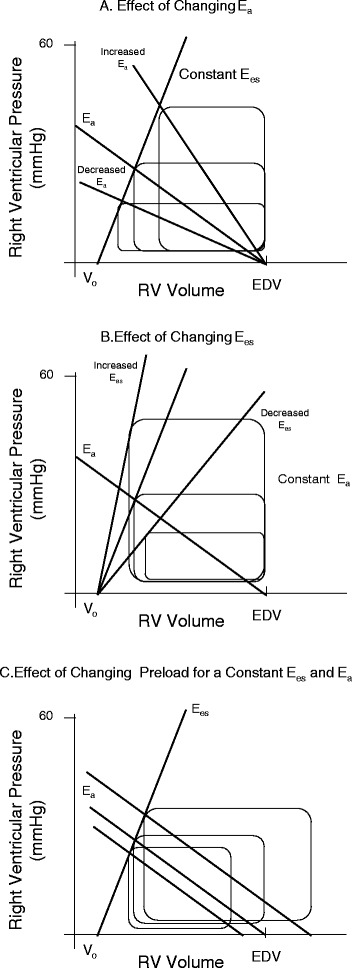
Right ventricular–pulmonary arterial coupling as described by the effects of changes in right ventricular (*RV*) end-systolic elastance (*Ees*), pulmonary arterial elastance (*Ea*) and RV end-diastolic volume on RV systolic pressure and stroke volume. **a** As Ea varies. **b** As Ees varies. **c** As end-diastolic volume varies

**Fig. 2:**
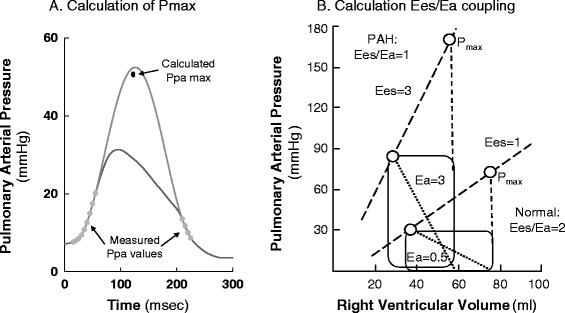
Single beat estimates of right ventricular–arterial coupling (*Ees* and *Ea*) using instantaneous pressure and flow measures. **a** Calculation of maximal isometric pulmonary arterial pressure (modified from Brimioulle et al. [43]). **b** Superimposition of right ventricular volume on pressures to calculate Ees and Ea for both a normal patient and one with pulmonary hypertension (*PAH*) (modified from Kuehne et al. [26])

RV contractility can be impaired by a variety of processes associated with increased pulmonary vascular resistance. For example, if RV dilation occurs, then systemic arterial hypotension can develop as LV end-diastolic volume is restricted. This systemic arterial hypotension must decrease right coronary blood flow, causing ischemic RV dysfunction. Similarly, there are many causes of increased pulmonary artery resistance that are treated differently. For example, pulmonary vascular resistance can increase due to pulmonary vasoconstriction (e.g., hypoxic pulmonary vasoconstriction treated by lung recruitment and supplemental oxygen), mechanical obstruction (e.g., pulmonary embolism treated by thrombolysis or thrombectomy), or extrinsic compression (e.g., hyperinflation treated by maneuvers that decrease hyperinflation). All of these processes can acutely impair RV performance. Similarly, patients with pre-existent chronic pulmonary diseases (e.g., chronic obstructive lung disease, pulmonary fibrosis, primary pulmonary hypertension) may also become acutely ill [50]. Thus, the assessment of RV to pulmonary arterial Ees/Ea coupling should be a useful tool in defining overall RV wellness and cardiovascular reserve (Fig. 1) [51]. Figure 1 illustrates how RV systolic pressure and stroke volume are tightly coupled to RV Ees, pulmonary Ea, and RV end-diastolic volume. As illustrated in Fig. 1, RV stroke volume can increase if either Ees or RV end-diastolic volume increases or Ea decreases and vice versa for conditions that cause RV stroke volume to decrease. Importantly, RV Ees/Ea can be assessed at the bedside with measures of pulmonary arterial pressure and echocardiography or other estimates of RV stroke volume [43] (Fig. 2). Thus, one can assess ventricular coupling at the bedside. Although with chronic pulmonary hypertension the right ventricle initially hypertrophies, increasing RV Ees to match the increased pulmonary arterial Ea, this is not possible with acute increases in pulmonary arterial Ea as often occurs in the critically ill.

The adaptive processes associated with chronic pulmonary hypertension have been extensively described. Chronic pulmonary hypertension induces RV hypertrophy. Early in the process, RV hypertrophy initially develops in the pulmonary outflow tract (infundibulum) [52] because this is the last region of the contracting RV to see increased pressures and thus will have the highest wall stress [53]. Assessment of RV strain in the setting of chronic pulmonary hypertension can be done using RV speckle tracking from echocardiographic images of the RV free wall once RV hypertrophy has developed [54]. Indeed, Vanderpool et al. [55] reported that impaired RV Ees/Ea is the most sensitive predictor of death or need for lung transplantation in patients with chronic pulmonary hypertension. RV adaption to pulmonary hypertension proceeds from minimal outflow tract hypertrophy with normal right atrial pressure, generalized RV hypertrophy with sustained elevated right atrial pressure, to end-stage dilated cardiomyopathy identical to end-stage LV failure. Since coronary blood flow does not increase, despite increased RV muscle mass, RV ischemia may occur if arterial pressure becomes less than pulmonary arterial pressure. Similarly, RV hypertrophy results in impaired lusotrophy (impaired diastolic relaxation); thus, tachycardia can induce marked increased right atrial pressures owing to this functional decrease in RV diastolic compliance. Finally, RV dilation with end-stage RV failure, when associated with hyperinflation, commonly seen in patients with chronic obstructive lung disease, will markedly impede LV filling owing to pericardial volume limitations [56], spiraling to cardiogenic shock.

Treatment priorities in the setting of chronic pulmonary hypertension are to pharmacologically reduce pulmonary vasomotor tone without causing concomitant systemic arterial hypotension with its associated detrimental decreased coronary perfusion and reflex tachycardia. Drugs such as sildenafil [57] and endothelin receptor blockers may be used. Fluid restriction and the cautious use of diuretics to find the sweet spot for RV filling should also be done if evidence of RV volume overload (e.g., paradoxical septal shift by echocardiography) are also present.

## Acute cor pulmonale

How RV Ees/Ea changes acutely in humans to increase in Ea has not been quantified but most likely is associated with impaired RV Ees, as the associated RV dilation and impaired LV filling and contraction coupled with systemic hypotension should impair intrinsic RV contractile function. In the naïve state, the right ventricle is designed to markedly alter its end-diastolic volume as needed to accommodate markedly changing rates of venous return that can vary by 200 % over a few heart beats. The primary factor limiting gross RV over-distention is the pericardium, as it limits absolute biventricular volumes [58]. Thus, sudden increases in RV end-diastolic volume, as may occur during deep spontaneous inspiratory efforts, will be associated with a matched decrease in LV end-diastolic volumes by the process of ventricular interdependence [59]. This then causes a ventilation-associated variation in systolic arterial pressure, known as pulsus paradoxus owing to the matched decreased LV stroke volume. However, if RV dilation is sustained because of impaired RV ejection due to increased outflow impedance, then acute reductions in LV output must occur, causing systemic hypotension, RV free wall ischemia, and worsening RV contractile function, dilating the RV further and decreasing further LV end-diastolic volume. This rapidly spiraling detrimental process can cause complete heart stand still and is presumably the cause of sudden death associated with massive pulmonary embolism [60]. The signs of acute cor pulmonale include increased right atrial pressure, tricuspid regurgitation, and RV dilation, such that RV end-diastolic mid-axis diameters equal or exceed LV end-diastolic diameters and paradoxical septal shift [61]. Importantly, pulmonary arterial pressure need not be elevated if the RV contraction is profoundly impaired, as the right ventricle under these circumstances cannot generate a sufficient pressure to cause pulmonary arterial pressure to increase.

The treatment of acute cor pulmonale is to reverse the cause of the increased pulmonary vascular resistance while maintaining an adequate mean arterial pressure. Systemic arterial tone can be supported either selectively (e.g., norepinephrine infusion) or generally (e.g., epinephrine infusion) as the cause of the cardiovascular collapse is often unclear. For massive pulmonary embolism thrombolysis, anti-coagulation [62] and, if severe and unresponsive, pulmonary thrombectomy should be considered early [63]. Selective pulmonary vasodilator agents (e.g., prostaglandins) may also be infused if an associated increased pulmonary vasomotor tone is suspected (e.g., anaphylaxis). Reversing pulmonary hyperinflation using bronchodilators and altering ventilator settings to maximize expiratory time and minimize tidal volume and using low tidal volume ventilation will help [64]. Importantly, fluid resuscitation is problematic. Rapid fluid infusion will worsen RV dilation and be detrimental whereas fluid restriction may limit venous return during a time when a higher upstream vascular pressure is needed to overcome the elevated right atrial pressure in driving venous return. There are no clear accepted guidelines for fluid resuscitation but all treatments can be guided by echocardiographic analysis [65]. A reasonable approach as suggested Vieillard-Baron et al. [66] is to allow the right ventricle to define its required volume by continuous assessment of RV volumes and performance by echocardiography. In an otherwise salvageable patient with unresponsive acute cor pulmonale the use of mechanical circulatory support with either a RV assist device or total artificial heart should be considered. Within this context, we suggested that sudden increases in RV end-diastolic pressure in response to fluid loading should also be a stopping rule for fluid infusion [28]. Deciding when to switch from completely aggressive medical to additional surgical options (thromobectomy or RV assist device insertion), if considered, should be done early as mortality increases with delay in therapy [67].

## Conclusions

RV function is not considered important for overall cardiovascular homeostasis until it is. Its primary role is to sustain an effective cardiac output by minimizing the impedance to systemic venous return to the heart while also not limiting LV filling. The right ventricle accomplishes this by keeping right atrial pressure as low as possible and having it not vary as venous return rate varies widely. To the extent that right atrial pressure increases, as may occur during fluid resuscitation, the use of mechanical ventilation, or disease, then maximal cardiac output responses to increased demand will be limited. In the extreme this will result in cardiogenic shock. The key factor regulating RV performance is pulmonary arterial impedance quantified by either pulmonary arterial pressure or pulmonary Ea. In cases where pulmonary arterial pressure increases acutely, causing acute cor pulmonale, the primary treatments are to increase systemic arterial pressure enough to sustain right coronary blood flow while attempting to revise the causes of increased pulmonary arterial pressure. In the setting of chronic pulmonary arterial hypertension, pharmacologic vasodilator therapy to prevent systemic hypotension and close fluid management to prevent both RV under- and overfilling are necessary. Central to the clinical assessment and management of RV failure are dynamic measures of right atrial pressure changes and bedside echocardiography, although other imaging methods may be useful in selected patients.

## Abbreviations

Ea, arterial elastance; Ees, end-systolic elastance; ITP, intrathoracic pressure; LV, left ventricular; PEEP, positive end-expiratory pressure; RV, right ventricular; Pmsa, mean systemic pressure analogue calaculated using Guyton physiologic assupptions; Ppa, pulmonary artery pressure
