# Erratum to: The right ventricle: interaction with the pulmonary circulation

**DOI:** 10.1186/s13054-016-1524-x

**Published:** 2016-11-10

**Authors:** Michael R. Pinsky

**Affiliations:** 1 Department of Critical Care Medicine, University of Pittsburgh, Pittsburgh, PA USA; 2 Department of Anesthesiology, University of California, East Campus Office Building, MC 7651, 9444 Medical Center Drive, Room 3-048, La Jolla, San Diego, CA 92093 USA

## Erratum

Unfortunately, the original article [1] contains an error in Fig. 2: “pulmonary” should be corrected to “right ventricular”, please find the correct Figure below.

**Fig. 2:**
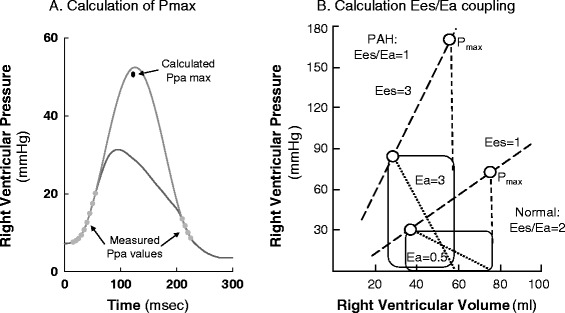
ᅟ
